# Linalool Activates Oxidative and Calcium Burst and CAM3-ACA8 Participates in Calcium Recovery in Arabidopsis Leaves

**DOI:** 10.3390/ijms23105357

**Published:** 2022-05-11

**Authors:** Chunyang Jiao, Junqing Gong, Zhujuan Guo, Shuwen Li, Yixin Zuo, Yingbai Shen

**Affiliations:** 1 College of Biological Sciences and Technology, Beijing Forestry University, Beijing 100083, China; chunyangjiao@126.com (C.J.); 13120030501@sohu.com (J.G.); gzj2190073@163.com (Z.G.); zhuzhuxia20220509@163.com (S.L.); zuoyx2020@163.com (Y.Z.); 2 National Engineering Laboratory for Tree Breeding, Beijing Forestry University, Beijing 100083, China

**Keywords:** linalool, H_2_O_2_ signaling, calcium signaling, *Plutella xylostella*, CAM3-ACA8

## Abstract

Plants produce linalool to respond to biotic stress, but the linalool-induced early signal remains unclear. In wild-type Arabidopsis, plant resistance to diamondback moth (*Plutella xylostella*) increased more strongly in a linalool-treated group than in an untreated control group. H_2_O_2_ and Ca^2+^, two important early signals that participated in biotic stress, burst after being treated with linalool in Arabidopsis mesophyll cells. Linalool treatment increased H_2_O_2_ and intracellular calcium concentrations in mesophyll cells, observed using a confocal microscope with laser scanning, and H_2_O_2_ signaling functions upstream of Ca^2+^ signaling by using inhibitors and mutants. Ca^2+^ efflux was detected using non-invasive micro-test technology (NMT), and Ca^2+^ efflux was also inhibited by NADPH oxidase inhibitor DPI (diphenyleneiodonium chloride) and in cells of the NADPH oxidase mutant *rbohd*. To restore intracellular calcium levels, Ca^2+^-ATPase was activated, and calmodulin 3 (CAM3) participated in Ca^2+^-ATPase activation. This result is consistent with the interaction between CAM7 and Ca^2+^-ATPase isoform 8 (ACA8). In addition, a yeast two-hybrid assay, firefly luciferase complementation imaging assay, and an in vitro pulldown assay showed that CAM3 interacts with the N-terminus of ACA8, and qRT-PCR showed that some JA-related genes and defense genes expressions were enhanced when treated with linalool in Arabidopsis leaves. This study reveals that linalool enhances H_2_O_2_ and intracellular calcium concentrations in Arabidopsis mesophyll cells; CAM3-ACA8 reduces intracellular calcium concentrations, allowing cells to resume their resting state. Additionally, JA-related genes and defense genes’ expression may enhance plants’ defense when treated with linalool.

## 1. Introduction

During plant defense, calcium functions as a crucial early signal. When plants perceive external signals of a pathogen attack, such as oral elicitors from insects or bacterial flagella, Ca^2+^ ions immediately flow into the cytoplasm from extracellular calcium stores (in the interstitial spaces between the cell wall and plasmids or between cell walls) or intracellular calcium stores (in the vacuole, Golgi apparatus, or endoplasmic reticulum) [1,2]. Once Ca^2+^ has completed its function as a second messenger, it is returned to the calcium stores via Ca^2+^-ATPases and the Ca^2+^/proton antiporter system [3]. H_2_O_2_, a reactive oxygen species (ROS) that also participates in many defense mechanisms of plants, has a close relationship with Ca^2+^. Ca^2+^ and H_2_O_2_ signals can be induced by exogenous polyamine and jasmonic acid (JA) treatment [4] and are also elicited by herbivore feeding [5]. Thus, Ca^2+^ and H_2_O_2_ participate in many resistance mechanisms in plants.

Ca^2+^ and H_2_O_2_ function together in two processes in plants: Ca^2+^-induced ROS production and ROS-induced Ca^2+^ release. There are three ways that Ca^2+^ induces ROS: Ca^2+^ activates RBOH by binding to the EF region of the RBOH protein in N-terminal; Ca^2+^-CPKs phosphorylates RBOH; Ca^2+^ induces the accumulation of phosphatidic acid (PA), which binds to the N-terminus of RBOH and activates RBOH. ROS-induced Ca^2+^ release is mainly caused by ROS directly activating or inhibiting the activity of calcium channels or pumps regulating the Ca^2+^ concentration [6]. Additionally, the sequence of calcium and ROS bursts may be an important factor for plants to recognize different stimulations.

Calmodulins (CAMs), including the seven CAMs encoded in the Arabidopsis (*Arabidopsis thaliana*) genome, play important roles in plant stress or defense responses [7]. AtCaM1 and AtCaM4 interact with *S*-nitroso glutathione reductase (GSNOR), an important component of nitric oxide (NO) homeostasis, during the salt-stress response, as revealed by protein–protein interaction assays [8]. Moreover, AtCAM4, which negatively affects freezing tolerance, interacts with the CAM-binding protein PATELLIN 1 (PATL1) to regulate Ca^2+^ signal transduction [9]. AtCAM3 regulates heat shock transcription factor (HSF) activity, HSP gene expression, and HSP protein accumulation [10]. Ca^2+^-CAM3 activates the expression of heat shock protein (HSP) genes in both the absence and presence of heat shock [11]. CAM7 was previously shown to interact with ACA8 [12]. Different CAMs have different functions, and the understanding of these CAMs’ functions needs further research.

When plants are exposed to insect herbivory, they emit volatile organic compounds (VOCs) [13,14]. The production of the terpene linalool, a fragrant volatile, is induced in plants in response to biotic stress. For example, linalool is among the many volatiles emitted by mossy sorrel (*Rumex confertus* Willd., *Polygonaceae*) when injured by weevils (*Hypera rumicis* L., *Coleoptera*: *Curculionidae*) [15]. Herbivory-induced linalool production increases plant resistance to insects. Chrysanthemum plants expressing the linalool synthase gene *FaNES1* produce more linalool than wild-type (WT) plants, which makes them smell better but taste worse to insects [16]. Similarly, compared to untreated control plants, rice (*Oryza sativa*) plants treated with linalool develop significantly shorter *Xoo* (*Xanthomonas oryzae* pv. *oryzae*) lesions. Notably, linalool enhances rice resistance to *Xoo* without having a direct antibacterial effect on *Xoo* [17]. The VOC linalool is emitted from plants attacked by insects and enhances plant resistance. However, few studies have focused on the early signal mechanisms induced by linalool. Although linalool enantiomers have different functions [18], in most cases, the two enantiomers are produced at the same time. Indeed, many studies have used a mixture of both enantiomers [19,20,21,22]. Thus, in the current study, we used a linalool mixture and did not separate the two enantiomers.

Here, using non-invasive micro-test technology (NMT) and confocal laser scanning microscopy, we demonstrated that linalool treatment enhances intracellular H_2_O_2_ and calcium concentrations in leaves. CAM3 then interacts with ACA8 to transfer Ca^2+^ out of the cytoplasm and reduce cytoplasmic calcium concentrations. Our findings uncover the role of linalool as a signal inducing H_2_O_2_ and calcium concentration changes in Arabidopsis leaves.

## 2. Results

### 2.1. Linalool Induces Defense Responses against P. xylostella in Arabidopsis

**Figure 1:**
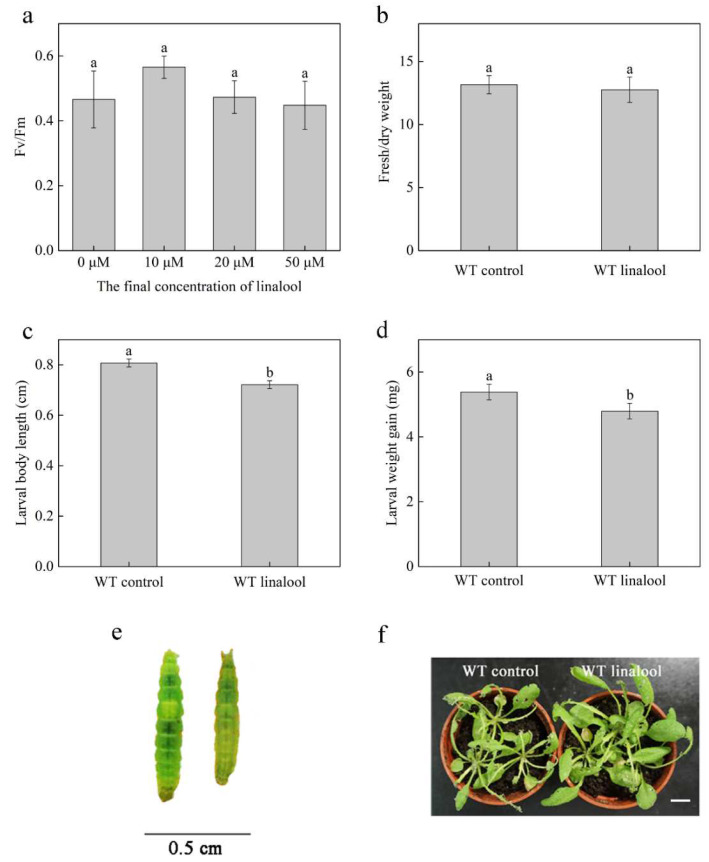
Arabidopsis plants show a linalool-induced plant defense against *P. xylostella.* (**a**) The maximal photochemical efficiency of Fv/Fm in different linalool treated groups. In the 0 μM control group, Arabidopsis plants were treated with ethyl alcohol. Additionally, in 10 μM, 20 μM, and 50 μM linalool groups, Arabidopsis plants were treated with linalool corresponding to the final concentrations. Error bars denote ± SEM, *n* = 6~8, *p* < 0.05, Dunnett’s C (variance not neat). (**b**) The ratio of fresh weight to dry weight. In the WT control group, Arabidopsis plants were treated with ethyl alcohol, and in the WT linalool group, Arabidopsis plants were treated with linalool at 20 μM final concentration. Error bars denote ± SEM (standard error of mean), *n* = 6~8, *p* < 0.05, Student’s *t*-test. (**c**,**d**) Larval body length and weight gain were measured 7 days after inoculation. Every pot had 3 Arabidopsis plants, 3 larvae were put in each plant, and each group (WT control and WT + linalool) contained approximately 25–40 larvae. Error bars denote ± SEM, *n* ≥ 25, and columns labeled with different letters are significantly different at *p* < 0.05, Student’s *t*-test. (**e**) Larvae phenotypes of the WT control (left) and WT + linalool (right). (**f**) Conditions of plants after 7 days of larval feeding. The plants were grown for 4 weeks (10 leaves). The scale bar represents 1 cm.

Determination of chlorophyll fluorescence is a common method of studying plant stress responses, particularly in photosystem II (PSII). Additionally, the maximal photochemical efficiency, Fv/Fm, shows the degree of damage in the PSII reaction centers [23]. We set up 10 μM, 20 μM, and 50 μM in three linalool final fumigation concentrations, and the three groups did not have significant differences in Fv/Fm compared with the 0 μM control group (use ethyl alcohol) after 24 h treatment (Figure 1a). This result indicated that there were no stresses on Arabidopsis plants in 10 μM, 20 μM, and 50 μM linalool groups. The Fv/Fm value increased slightly in the lowest linalool concentration group, the 10 μM linalool group (about 0.57), when compared with that in the 0 μM control group (about 0.47). Additionally, the 20 μM linalool group (about 0.47) and 50 μM linalool group (about 0.45) were closer to the 0 μM control group. Thus, we selected the second linalool concentration, 20 μM. In addition, the fresh/dry weight ratio can also reflect the growth status of plants. We detected the fresh/day weight in the WT control group (use ethyl alcohol) and WT linalool group (20 μM linalool final fumigation concentration). Additionally, there was no significant difference between the two groups (Figure 1b). The two experiments’ results indicated that the 20 μM linalool final fumigation concentration did not affect the growth of Arabidopsis plants and could be used for subsequent experiments.

To confirm that linalool induces resistance to *P. xylostella* in Arabidopsis, we inoculated WT plants with *P. xylostella* larvae after 24 h of linalool treatment; the control group was treated with ethyl alcohol. After 7 days, we measured the length and weight of *P. xylostella* larvae. Both growth indicators were significantly lower in linalool-treated plants than in control plants (Figure 1c–e). In addition, the plant leaf damage was also more severe in the control group than in the linalool group (Figure 1f). These results indicate that linalool induces Arabidopsis plant defense against *P. xylostella*. Early signals are the first response of plants to external stimuli [24]. To further investigate the early signals, we conducted the following experiments.

### 2.2. Linalool Increases H_2_O_2_ Levels in Arabidopsis Mesophyll Cells

**Figure 2:**
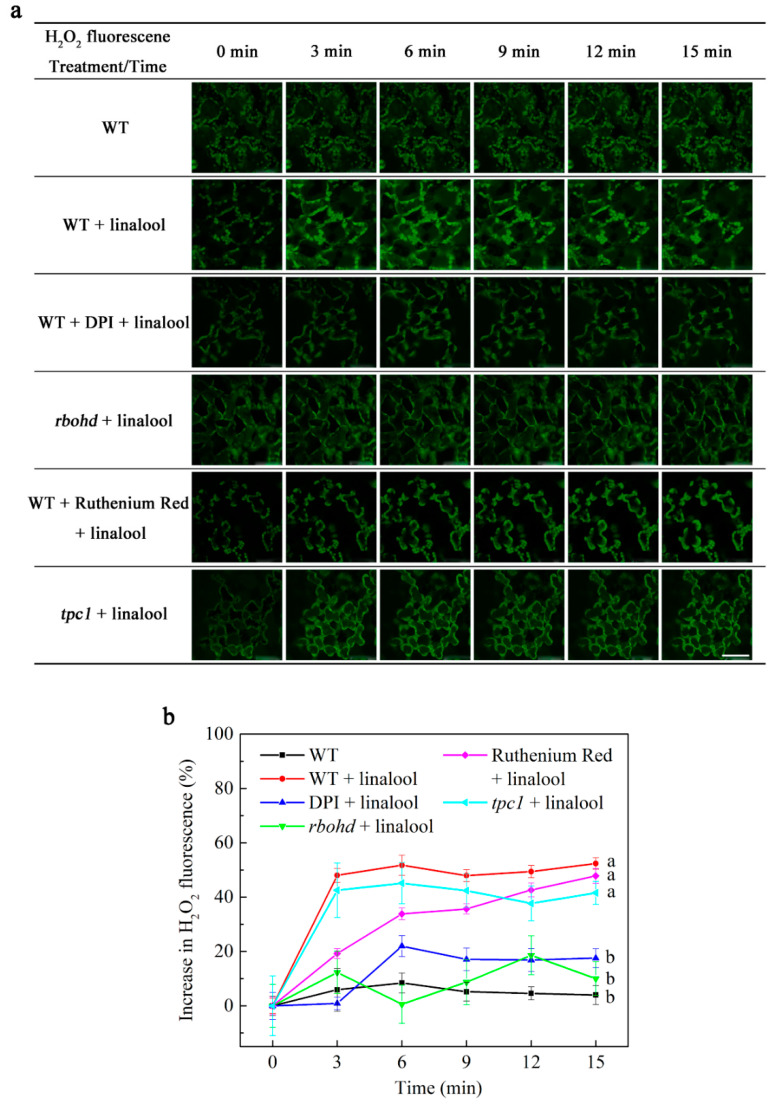
Linalool-induced increases in H_2_O_2_ levels depend on RBOHD but not on internal Ca^2+^ release channels in Arabidopsis mesophyll cells. (**a**) The cells were washed to remove 50 μM H_2_DCF-DA test solution (0 min), then linalool was added, and the cells were photographed every 3 min. The scale bar represents 50 μm. (**b**) Analysis of changes in H_2_O_2_ fluorescence indicated that increases in H_2_O_2_ fluorescence were significantly suppressed in DPI-treated and *rbohd* cells compared to the WT + linalool-treated group but were unaffected in ruthenium-red-treated and *tpc1* cells. The plants were grown for 2 weeks. Error bars denote ± SEM, *n* ≥ 20, and means labeled with different letters are significantly different at *p* < 0.05, Dunnett’s C (variance not neat).

H_2_O_2_ functions as an early signal in various signal transduction processes. We tested the variation in H_2_O_2_ levels in Arabidopsis mesophyll cells by performing confocal laser scanning microscopy of mesophyll cells from linalool-treated versus untreated control plants. H_2_O_2_ levels were strongly increased in mesophyll cells from linalool-treated WT plants compared to the control. Following linalool treatment, we photographed the plants every 3 min for 15 min (Figure 2a). High levels (increase about 50% above control) of H_2_O_2_ were maintained in the WT linalool group throughout the 15 min period (Figure 2b). In contrast, in linalool-treated mesophyll cells pretreated with the NADPH oxidase inhibitor DPI (diphenyleneiodonium chloride) and in cells of the NADPH oxidase mutant *rbohd*, H_2_O_2_ enhanced levels (about 20% above control) were lower than WT linalool group after linalool treatment (Figure 2b). H_2_O_2_ levels (about 30–45% above control) in linalool-treated WT cells pretreated with ruthenium red (a Ca^2+^ channel inhibitor that blocks the release of internal Ca^2+^) [25,26] and *tpc1* cells were similar to those of the WT linalool group (Figure 2b; data from controls are shown in Figure S1). These results indicate that RBOHD functions in linalool signal transduction and that changes in calcium concentrations have little effect on H_2_O_2_ levels in mesophyll cells.

### 2.3. Linalool Increases Intracellular Calcium Levels in Arabidopsis Mesophyll Cells and Functions Downstream of H_2_O_2_ Signaling Burst

**Figure 3:**
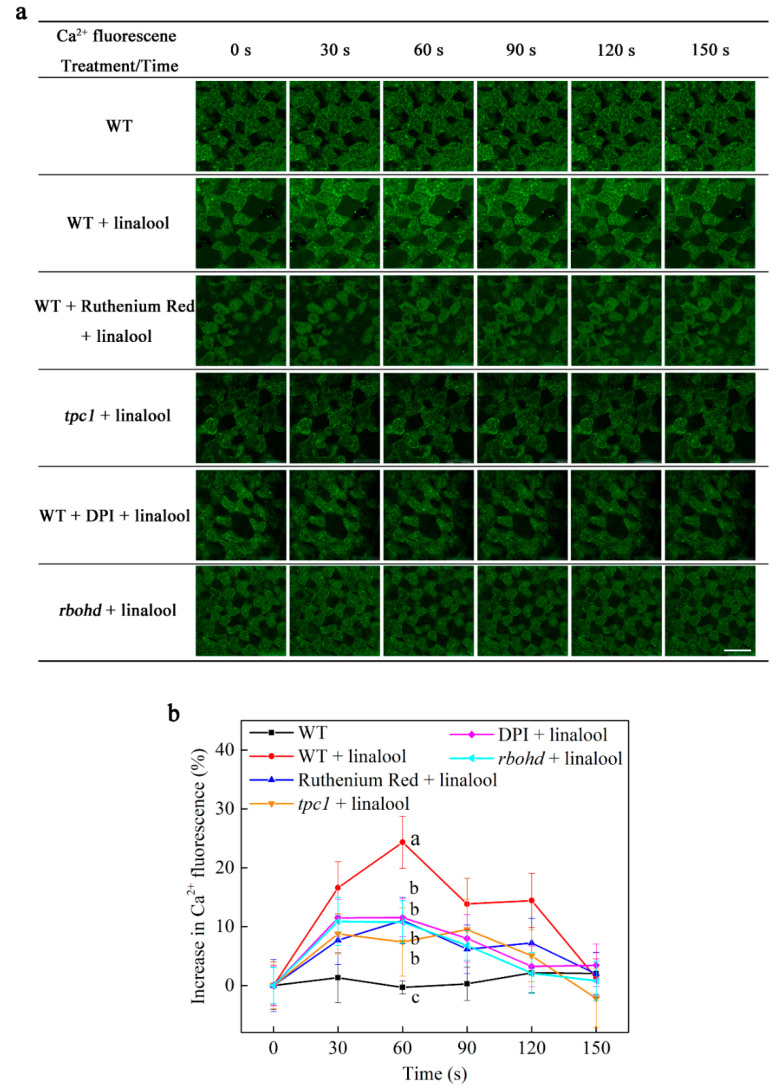
Linalool-induced increases in intracellular calcium levels depend on RBOHD and internal Ca^2+^ release channels in Arabidopsis mesophyll cells (**a**) The cells were washed to remove the 10 μM Fluo3-AM test solution (0 min), linalool was added, and the cells were photographed every 30 s. The scale bar represents 50 μm. (**b**) Linalool-induced increases in Ca^2+^ fluorescence were significantly suppressed in ruthenium-red-treated WT, *tpc1*, DPI-treated WT, and *rbohd* cells compared to linalool-treated WT control cells. The plants were grown for 2 weeks. Error bars denote ± SEM, *n* ≥ 20, and means labeled with different letters are significantly different at *p* < 0.05, Dunnett’s C (variance not neat).

To investigate the changes in intracellular calcium concentrations, we performed confocal laser scanning microscopy to detect Ca^2+^ fluorescence. Ca^2+^ fluorescence was higher in the WT linalool group versus the other groups from 30 s to 120 s (Figure 3a; data for the control groups are shown in Figure S2). We then calculated the increase in Ca^2+^ fluorescence. When WT Arabidopsis mesophyll cells were treated with linalool, intracellular Ca^2+^ fluorescence rapidly increased for 30 s (about 17% above control), remained at a similar level at 60 s, and then gradually decreased to the initial level (Figure 3b). However, in ruthenium-red-treated WT cells, *tpc1* cells, DPI-treated WT cells, and *rbohd* cells, only slight increases (about 8–10% above control) in calcium concentrations were detected during this period in response to linalool treatment (Figure 3a,b). These results suggest that linalool increases calcium concentrations in Arabidopsis mesophyll cells due to the influx of calcium from internal calcium stores. The results of analysis of H_2_O_2_ levels and variations in calcium concentrations in DPI-treated and *rbohd* cells suggest that H_2_O_2_ signaling burst functions upstream of the Ca^2+^ concentration increase.

**Figure 4:**
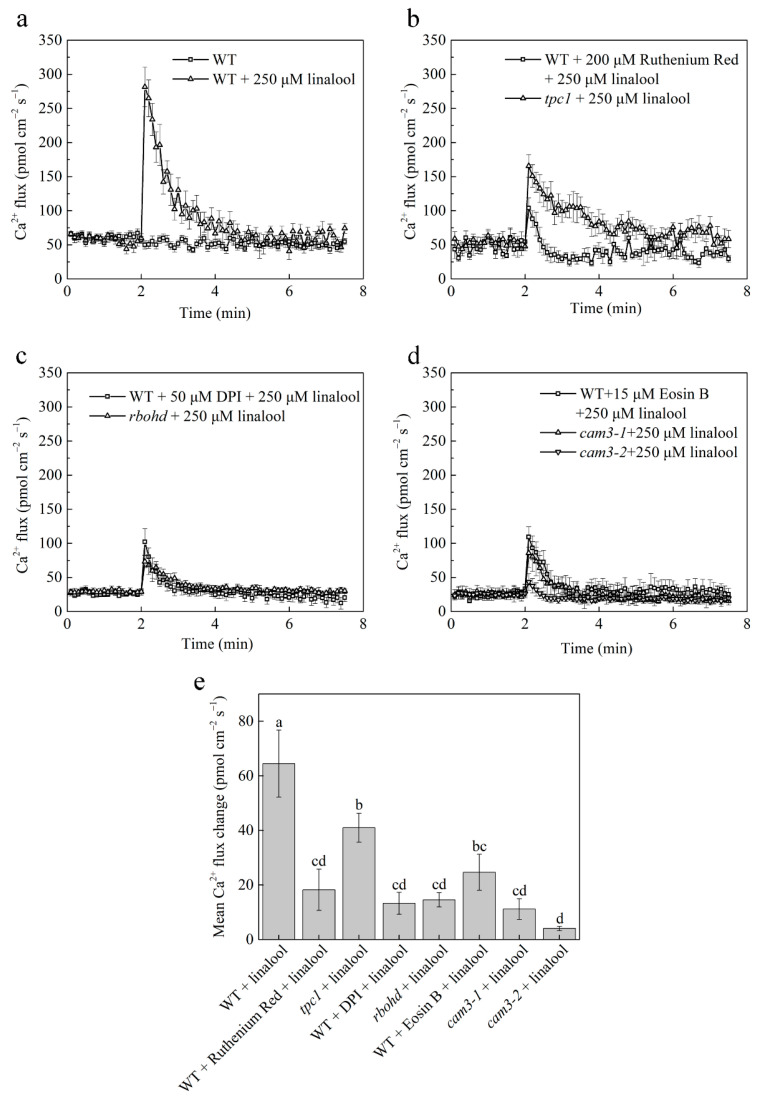
Calcium channel activity (related to changes in intracellular calcium levels) is induced by linalool treatment in Arabidopsis mesophyll cells. (**a**–**d**) Linalool treatment increases Ca^2+^ efflux. Ca^2+^ efflux was significantly suppressed in WT cells pretreated with ruthenium red and in *tpc1*, DPI-pretreated WT, *rbohd*, Eosin B-pretreated WT, and *cam3* cells. (**e**) A comparison of the peaks in mean Ca^2+^ efflux among groups shows that Ca^2+^ efflux was significantly suppressed in all groups compared to the linalool-treated WT control. The plants were grown for 2 weeks. Error bars denote ± SEM, *n* ≥ 6, and columns labeled with different letters are significantly different at *p* < 0.05, Dunnett’s C (variance not neat).

To further explore the changes in calcium flux in Arabidopsis mesophyll cells, we detected calcium flux by NMT. The test period was divided into pre-linalool treatment (pre), the linalool-responsive peak (peak), and the post-linalool response (post) based on the transient processing of Ca^2+^ ions by linalool. The pre-period lasted for 2 min, and the peak period began in the second minute. In WT mesophyll cells, Ca^2+^ flux peaked at 281 pmol cm^−2^ s^−1^ and then underwent a steady decline. By 2.5 min, Ca^2+^ flux was stable and entered the post-period. During both the pre- and post-periods, Ca^2+^ flux was approximately 58 pmol cm^−2^ s^−1^ (Figure 4a). When WT plants were pretreated with ruthenium red, the peak (104 pmol cm^−2^ s^−1^) was significantly reduced following linalool treatment compared to that in WT plants treated with linalool but not pretreated with ruthenium red. The peak of the *tpc1* mutant (166 pmol cm^−2^ s^−1^) showed the same degree of decline as that of the WT ruthenium red group (Figure 4b). In both DPI-pretreated (102 pmol cm^−2^ s^−1^) and *rbohd* (73 pmol cm^−2^ s^−1^) mesophyll cells, the peak following linalool treatment was reduced to a level similar to that of the WT ruthenium red and *tpc1* groups (Figure 4c). To further test the differences among these groups, we calculated the mean Ca^2+^ flux, finding that the peak was significantly higher in the WT linalool group than in the other groups. The mean Ca^2+^ flux was calculated and compared amongst experimental treatments to test the statistical differences among these groups (Figure 4e). The results showed that the mean Ca^2+^ fluxes were reduced in the ruthenium red pretreated group, *tpc1* mutant group, DPI-pretreated group, and *rbohd* mutant group. The result of Ca^2+^ flux is consistent with that of calcium concentrations.

### 2.4. CAM3 Interacts with ACA8 to Transport Calcium Ions Outside of Mesophyll Cells

After the calcium signal is transferred, the calcium must be transported into extracellular or intracellular calcium stores. Since the overall transmembrane calcium flow rate of the cell population at the monitoring site where the electrode monitored by NMT is located is efflux, we speculated that Ca^2+^-ATPase might play an important role in this process. We pretreated WT leaves with the Ca^2+^-ATPase inhibitor Eosin B and examined calcium flux. The peak of calcium flux was lower in the Eosin B-pretreated WT + linalool group (about 109 pmol cm^−2^ s^−1^) than in the WT + linalool group (about 281 pmol cm^−2^ s^−1^) (Figure 4d), indicating that Ca^2+^-ATPase functions in calcium transport. ACA8, a Ca^2+^-ATPase, is located in the plasma membrane. Additionally, CAM7 was previously shown to interact with ACA8 [12]. Because *CAM3* and *CAM7* are CAM family members that share high sequence similarity (up to 98.9%), the peak of calcium flux was also about 100 pmol cm^−2^ s^−1^ in the *cam3-1* + linalool group and about 19 pmol cm^−2^ s^−1^ in the *cam3-2* + linalool group (Figure 4d). We predicted that CAM3 is also a binding partner of ACA8. To investigate this hypothesis, we conducted Y2H, LCI, and in vitro pulldown assays.

**Figure 5:**
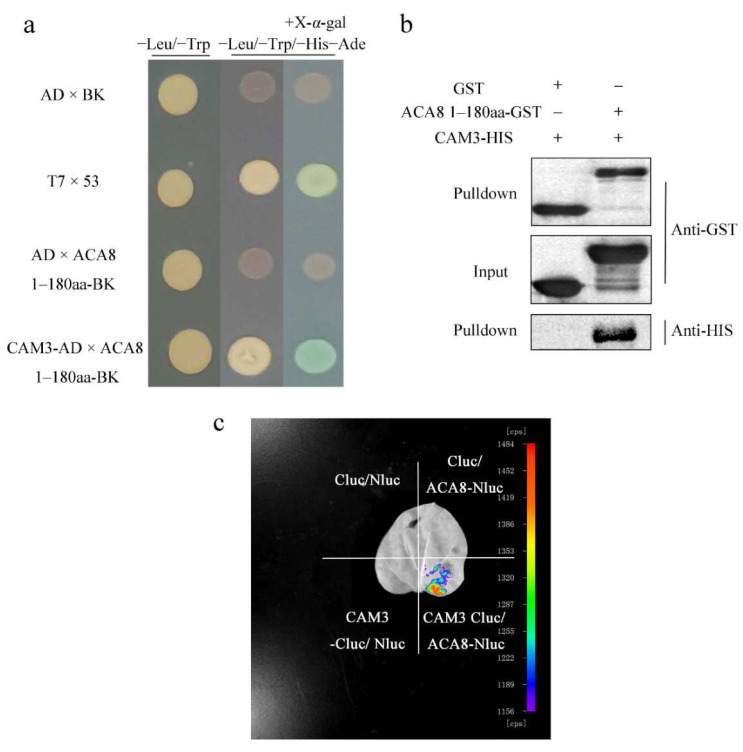
CAM3 interacts with ACA8. (**a**–**c**) Y2H, in vitro pulldown, and LCI assays show that CAM3 interacts with ACA8. (**a**,**b**) In the Y2H and in vitro pulldown assays, because ACA8 is a membrane protein, the N-terminus (residues 1–180), an intracellular domain, was used to interact with CAM3. (**c**) *N. benthamiana* leaves were divided into four parts: the bottom right part was an experimental group, and the others were control groups. Red represents the maximum fluorescence intensity.

Since ACA8 is a transmembrane protein, and CAM7 interacts with the N-terminus of ACA8, we used the N-terminus (residues 1–180) of ACA8 as bait and CAM3 as the prey protein, finding that the competent yeast strain AH109 cells with co-transformed plasmid pairs CAM3-AD plus ACA8 (residues 1–180)-BK grew on SD/−Leu/−Trp and SD/−Ade/−His/−Leu/−Trp and grew blue colonies on SD/−Ade/−His/−Leu/−Trp with X-α-gal. This result indicated that CAM3 binds to the N-terminus of ACA8 in the Y2H assay (Figure 5a). CAM3 also interacted with the N-terminus of ACA8 in a pulldown assay. In the anti-HIS pulldown line, CAM3–HIS was not detected in CAM3-HIS with GST group, but in CAM3-HIS with ACA8-GST group, CAM3-HIS was detected by immunoblot analysis using anti-HIS antibodies (Figure 5b). In LCI assay, *N. benthamiana* leaf parts injected with CAM3-Cluc/ACA8-Nluc showed stronger fluorescence than the other three (control) leaf parts (Cluc/Nluc, CAM3-Cluc/Nluc, and Cluc/ACA8-Nluc) (Figure 5c). These results demonstrate that CAM3 interacts with ACA8. In addition, we examined calcium flux in the *cam3* mutant after linalool treatment and found that the peak flux was lower in *cam3* than in WT plants following linalool treatment (Figure 4d). These results provide further evidence that CAM3 interacts with ACA8 to participate in calcium efflux.

### 2.5. Linalool Activates JA-Related Gene Expression

**Figure 6:**
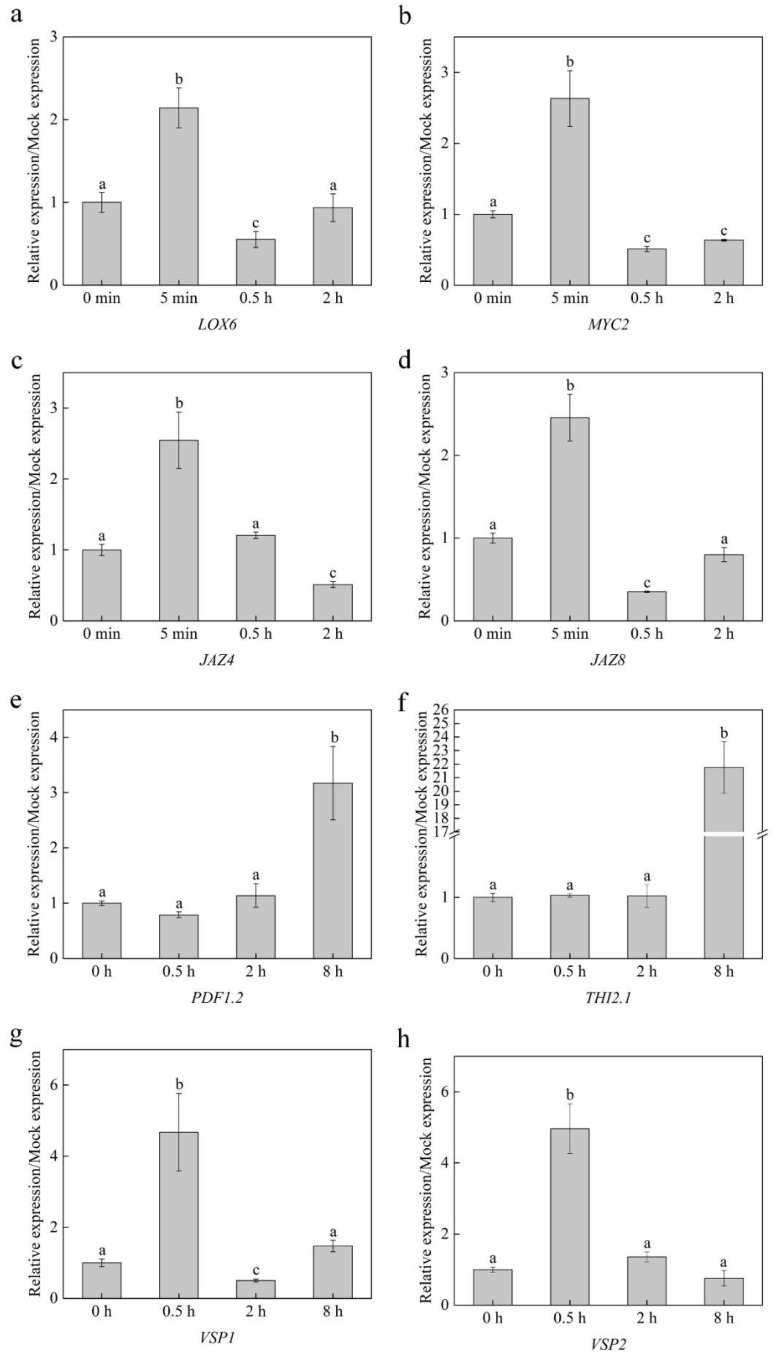
JA-related gene and defense gene expression induced by linalool. (**a**–**d**) JA-related gene expression in two-week-old Arabidopsis leaves after linalool treatment. The treated times were 5 min, 0.5 h, and 2 h. (**e**–**h**) Defense gene expression in two-week-old Arabidopsis leaves after linalool treatment. The treated times were 0.5 h, 2 h, and 8 h. Each group has three replicates. Every replicate includes dozens of seedlings (*n* ≥ 30), and error bars represent standard error; columns labeled with different letters are significantly different at *p* < 0.05, Dunnett’s C (variance not neat).

JA is an important hormone that functions in plant defense. To further explore the roles of JA in linalool-induced signaling, we measured the expression of important genes related to JA pathways, including *LOX6*, *MYC2*, *JAZ4*, and *JAZ8,* in WT plants following linalool treatment for 5 min, 0.5 h, and 2 h. In addition, we measured the expression of defense genes *PDF1.2*, *THI2.1*, *VSP1*, and *VSP2* in WT plants following linalool treatment for 0.5 h, 2 h, and 8 h. Almost all genes are expressed in the WT (Figure 6), as revealed by qPCR. The maximum value of JA-related gene expression was about 2~3-fold at 5 min (Figure 6a–d). In contrast, the maximum value of defense gene expression was not at the same time. The maximum value of *PDF1.2* (about 3.2-fold) and *THI2.1* (about 22-fold) gene expression was at 8 h (Figure 6e,f), and the maximum value of *VSP1* (about 4.7-fold) and *VSP2* (about 5-fold) gene expression was at 0.5 h (Figure 6g,h). Overall, these results indicate that linalool can induce JA-related gene and defense gene expression.

## 3. Discussion

In this study, we demonstrated that linalool, a volatile released from plants, enhances H_2_O_2_ and intracellular calcium concentrations in Arabidopsis mesophyll cells; CAM3-ACA8 reduces intracellular calcium concentrations, allowing cells to resume their calcium resting state. In addition, the observation that linalool treatment enhances plant defense may be due to JA-related genes’ expression and defense genes’ expression.

When Arabidopsis recognizes linalool, H_2_O_2_ levels in leaf mesophyll cells rapidly increase. We found that this H_2_O_2_ burst was inhibited in the leaves of WT plants pretreated with the NADPH oxidase inhibitor DPI and in leaves of the NADPH oxidase mutant *rbohd* (Figure 2). Similarly, linalool treatment resulted in rapid increases in the intracellular Ca^2+^ concentration and Ca^2+^ flux, and both of these effects were inhibited in DPI-treated and *rbohd* mesophyll cells (Figure 3 and Figure 4). Increases in intracellular Ca^2+^ concentrations and Ca^2+^ flux were also inhibited when we used the intracellular calcium store inhibitor ruthenium red and in *tpc1*, a mutant of TPC1 (a depolarization-activated Ca^2+^ channel located in the vacuolar membrane). Therefore, we measured H_2_O_2_ levels in ruthenium-red-pretreated wild-type and *tpc1* leaves following linalool treatment. The H_2_O_2_ burst was not inhibited in these cells, indicating that the linalool-induced H_2_O_2_ burst occurs before the increase of Ca^2+^ levels in Arabidopsis leaf mesophyll cells. (*E*)-2-hexenal can also increase the Ca^2+^ concentration in leaves of Arabidopsis thaliana. When leaves were pretreated with ruthenium red, calcium ions could not be induced by (*E*)-2-hexenal, suggesting that intracellular calcium store is involved in the increase in the (*E*)-2-hexenal-induced intracellular Ca^2+^ concentration [27].

Ca^2+^ and H_2_O_2_ function together in two processes in plants: Ca^2+^-induced ROS production and ROS-induced Ca^2+^ release [6]. Here, we demonstrated that linalool-induced Ca^2+^ and H_2_O_2_ signaling function in ROS-induced Ca^2+^ release. The recognition of different stimuli occurs via different processes during early signaling, making the sequence of early signaling events a special ’language’ in plant resistance.

TPC1 activation is regulated by many factors. Ca^2+^ binding to the TPC1 cytosolic EF-hand domain triggers conformational changes to activate TPC1 [28]. Additionally, ABA, ATP, cAMP, and Ca^2+^ treatment increase vacuolar Ca^2+^ release, while vacuolar Ca^2+^ efflux was strongly suppressed by H_2_O_2_ in an earlier study [29]. In our study, linalool-induced H_2_O_2_ burst is upstream of the enhancement of calcium concentration in Arabidopsis mesophyll cells. Therefore, there may be other ways to slightly increase intracellular calcium concentration in the cytoplasm before linalool-induced calcium is released from the intracellular calcium store, thereby activating TPC1 and releasing large amounts of calcium from the intracellular calcium store into the cytoplasm. At the same time, a more detailed study of how TPC1 is activated with linalool treatment should be undertaken in a separate investigation.

Ca^2+^ plays an important role in many signaling transduction processes, but high concentrations of calcium in the cytoplasm are toxic to cells. After calcium has functioned as a second messenger, it is transported into calcium stores. The P-type ATPase, Ca^2+^-ATPase, located in the plasma membrane and the intracellular calcium store membrane, participates in the process of calcium efflux. Studies have shown that only II B Ca^2+^-ATPase can bind to CAMs. When the N-terminal of Ca^2+^-ATPase binds to Ca^2+^, CAM also binds to the N-terminal CAM binding site [30]. By examining Ca^2+^ flux, we showed that linalool-treated mesophyll cells exhibit strong Ca^2+^ efflux. Therefore, we investigated whether the Ca^2+^-ATPase inhibitor Eosin B would inhibit this Ca^2+^ efflux. Our findings indicate that Ca^2+^-ATPase participates in linalool-induced Ca^2+^ efflux in mesophyll cells and that Ca^2+^ efflux is inhibited in *cam3* plants (Figure 4d). Since CAM7 interacts with ACA8 [12], *CAM3* and *CAM7* share high sequence similarity (up to 98.9%), so we suggested that CAM3 may also interact with ACA8. Y2H, LCI, and in vitro pulldown assays indicated that CAM3 binds to the N-terminus of ACA8 and functions in the Ca^2+^ efflux process (Figure 4d and Figure 5).

Early signals are the beginning of a series of responses that begin in plants when they feel external stimulations [24]. Plants that receive VOCs influence gene regulation, metabolism, phenotype, responses to stress, and behavioral choices [31]. In our study, linalool-treated plants can be seen as ‘receivers’ that change in gene regulation and responses to stress (linalool-treated plants are more resistant to insects) to improve the ability of plants to cope with biotic stress. In WT plants treated with linalool, the expression of almost all genes related to the JA pathway and defense genes increased compared to those in the untreated control (Figure 6). These results indicate that the linalool-induced upregulation of JA-related genes and defense genes made plants in a more stress-resistant state to enhance plant defense.

Notably, changes in intracellular Ca^2+^ concentrations regulate the JA biosynthesis pathway. When plants are wounded by insects, CML37 and CML42 influence the JA biosynthesis pathway [32,33]. Further studies could uncover the Ca^2+^ sensors involved in linalool-induced plant defense. In addition, much remains to be learned about the messages encoded in different VOCs. This knowledge could lay the foundation for the strategies plants use to defend themselves against insects.

## 4. Materials and Methods

### 4.1. Plant Materials and Culture Conditions

Wild-type (Col-0) Arabidopsis, *rbohd* CS9555 (AT5G47910), *tpc1* SALK_125658 (AT4G03560), *cam3-1* SALK_149754C (AT3G56800), and *cam3-2* SALK_042391C (AT3G56800) mutants were used in this study. The seeds were vernalized at 4 °C for 2 days in the dark. After vernalization, the seeds were surface-sterilized for 4 min in 75% ethyl alcohol, washed four times in sterile water, sown in the autoclaved soil mixture, and placed in an incubator (Percival model: I-36vl). Plants were grown in soil at 21–23 °C and 70% relative humidity with a light intensity of 80–110 μmol m^−2^ s^−1^ under long-day (16 h light/8 h dark) conditions. The plants used for insect inoculation were grown for 4 weeks, and the plants used in the other experiments were grown for 2 weeks before treatment.

### 4.2. Plutella xylostella Egg Hatching and Inoculation

*Plutella xylostella* (diamondback moth) eggs were hatched in an incubator at 25 ± 1 °C with a relative humidity of 50 ± 10%. After 1–2 days, larvae that were identical in length and weight were chosen for inoculation onto Arabidopsis plants; each plant was inoculated with three larvae. WT control and WT linalool-treated plants were prepared for analysis. Seven days later, the insects were removed, and their lengths and weights were measured. Each group contained 25–40 insects.

### 4.3. Chlorophyll Fluorescence Measurement

WT control and WT linalool-treated plants were used to measure leaf chlorophyll fluorescence. After 0.5 h dark adaption, the maximum photochemical efficiency Fv/Fm was of PSII, obtained with a modulated fluorometer IMAGING PAM (Zealquest Scientific Technology Co., Ltd., Shanghai, China). Every group contained 6–8 Arabidopsis plants.

### 4.4. Linalool Fumigation

Arabidopsis plants were treated with linalool (≥97%, a mixture of the two enantiomers of linalool, purchased from Sigma-Aldrich, Shanghai, China, CAS NO.: 78-70-6) via fumigation in glass bell jars (height: 12.5 cm; diameter: 15 cm) in insect growth test. Cotton balls 1 cm in diameter were soaked in linalool dissolved in ethyl alcohol (in insect inoculation and qRT-PCR experiments; linalool’s final concentration was 20 μM) and hung in the bell jars; cotton balls soaked in the same volume of ethyl alcohol were used for the control. After adding the cotton balls, the bell jars were immediately sealed with Vaseline petroleum jelly.

### 4.5. Fresh/Dry Weight Measurements

Arabidopsis plants treated with ethyl alcohol and linalool (linalool’s final concentration was 20 μM) were used to measure fresh weight. Additionally, the samples were then dried in an oven at 80 °C for 24 h to constant weight and weighed as dry weight. Every group contained 6–8 Arabidopsis plants.

### 4.6. Ca^2+^ Flux Measurements in Mesophyll Cells

Ca^2+^ flux in mesophyll cells was measured using non-invasive micro-test technology (NMT) (BIO-001A, Younger, MA, USA). Before testing, a small cut was made in the leaves of 2-week-old Arabidopsis plants. The leaves were soaked in test solution (0.1 mmol L^−1^ KCl, 0.1 mmol L^−1^ CaCl_2_, 0.1 mmol L^−1^ MgCl_2_, 0.5 mmol L^−1^ NaCl, 0.3 mmol L^−1^ MES, 0.2 mmol L^−1^ Na_2_SO_4_, pH 6.0) for approximately 30 min. Silanized glass micropipettes (2–4 μm aperture) were filled with electrolyte solution (100 mmol L^−1^ CaCl_2_) and front-filled with a selective liquid ion exchange (LIX) cocktail to approximately 10 μm. The glass micropipettes were connected to the NMT system with a silver chloride wire, and electrodes with Nernstian slopes of 28 ± 5 mV log^−1^ were used.

The Ca^2+^ concentration in the mesophyll cells was measured near the cells and 30 μm away from the cells at 0.2 Hz. Each leaf was tested for 2 min before linalool treatment (pre-linalool treatment; pre). After linalool was quickly added to the test solution to a final concentration of 100 μM, data were collected for approximately 2.5 min as the linalool response peak (peak) group. Data were then collected from the post-linalool response (post) group to indicate the end of the reaction. The final flux values are reported as the mean of eight individual plants per treatment. The Ca^2+^ flux was calculated as:

(1) $$J=-D\frac{\Delta C}{\Delta X}$$

where *J* is the flux of Ca^2+^ (pmol cm^−2^ s^−1^), D is the diffusion coefficient (0.79 × 10^−5^ cm^2^ s^−1^), Δ*C* is the difference between the concentrations near and far from the cells, and Δ*X* is 30 μm. Each group contained 6–8 replicates.

### 4.7. Ca^2+^ Fluorescence Measurements in Mesophyll Cells

Before treatment with Fluo3-AM (10 μM), Arabidopsis leaves were soaked in a test solution (the same solution used for the Ca^2+^ flux experiment). The leaves were then soaked in Fluo3-AM solution to label Ca^2+^ ions for 1 h in the dark in an incubator. The leaves were washed and placed in the fresh test solution. Ca^2+^ fluorescence was detected under a confocal laser scanning microscope (Leica TCS SP8, Leica Microsystems, Wetzlar, Germany). The excitation wavelength was 488 nm, and the emission wavelength was 530 nm. Before linalool (a final concentration of 100 μM) addition (0 min), the basal level of Ca^2+^ fluorescence in mesophyll cells was measured. Following the addition of linalool, fluorescence was measured every 30 sec for 150 s. Each group contained 20–40 cells.

The increase in Ca^2+^ fluorescence = (*X* − *a*)/*a* × 100%, where *X* is the fluorescence of linalool-treated samples, and *a* is fluorescence at 0 s. The value for each group was calculated separately.

### 4.8. H_2_O_2_ Fluorescence Measurements in Mesophyll Cells

H_2_DCF-DA was used to measure changes in H_2_O_2_ levels in Arabidopsis mesophyll cells. Following incubation in the test solution, the leaves were incubated in 50 μM H_2_DCF-DA for 15 min. Confocal laser scanning microscopy (Leica TCS SP8, Leica Microsystems, Wetzlar, Germany) was used to detect H_2_O_2_ fluorescence at an excitation wavelength of 488 nm and an emission wavelength of 510–530 nm. H_2_O_2_ fluorescence measurements were obtained at 0 min (before the addition of linalool) and every 3 min for 15 min. The increase in H_2_O_2_ fluorescence was calculated as Ca^2+^, and linalool’s final concentration was 100 μM. Each group contained 20–40 cells.

### 4.9. Yeast Two-Hybrid (Y2H) Assay

The sequences encoding CAM3 were cloned into pGADT7, and the sequences encoding amino acid residues 1–180 of ACA8 (ACA8 (residues 1–180)) were cloned into pGBKT7, respectively. The plasmid pairs CAM3-AD plus ACA8 (residues 1–180)-BK were co-transformed into competent yeast strain AH109 cells. The cells were grown on SD/−Leu/−Trp medium. After 4 days, growing colonies were transferred to SD/−Ade/−His/−Leu/−Trp for further verification. Growing colonies on SD/−Ade/−His/−Leu/−Trp were transferred to SD/−Ade/−His/−Leu/−Trp with X-α-gal. Cell growth on SD/−Leu/−Trp and SD/−Ade/−His/−Leu/−Trp and blue colonies on SD/−Ade/−His/−Leu/−Trp with X-α-gal indicate protein–protein interactions between the two proteins.

### 4.10. Firefly Luciferase Complementation Imaging Assay (LCI)

*CAM3* was cloned into the Cluc plasmid, and *ACA8* was cloned into the Nluc plasmid, respectively. Additionally, the plasmids were transformed into Agrobacterium strain GV3101, respectively. Fluorescence from luciferase in *Nicotiana tabacum* leaves infected with pairs of Agrobacterium was imaged with a molecular imaging system (LB983, Berthold Technologies, Bad Wildbad, Germany). Each leaf was divided into four quadrants before injection with the following combinations: Cluc/Nluc, CAM3-Cluc/Nluc, Cluc/ACA8-Nluc, and CAM3-Cluc/ACA8-Nluc, respectively.

### 4.11. In Vitro Pulldown Assay

The genes encoding ACA8 (residues 1–180) were cloned into pGEX-4T-1, and the genes encoding CAM3 were cloned into pET28A. ACA8-GST and CAM3-HIS were transformed into *E. coli* Rosetta (DE3) cells for protein expression. Protein-GST was used as the bait protein. Glutathione beads containing 50 µg ACA8-GST or GST were incubated with 50 µg CAM3-HIS in pulldown buffer (1% NP40, 150 mM NaCl, 50 mM Tris–HCl, 1 mM EDTA, pH 7.5) at 4 °C for 2 h, respectively. The protein beads complexes were washed with pulldown buffer, then centrifuged 500× *g* for 5 min, the supernatant was removed, and the whole process was repeated 4 times; at last, the SDS sample buffer was boiled for 5–10 min. The bindings of CAM3-HIS with ACA8-GST were detected by immunoblot analysis using anti-GST and anti-HIS antibodies.

### 4.12. qRT-PCR

For quantitative RT-PCR, total RNA was extracted from the samples using an RNA extraction kit, and cDNA was generated using a reverse transcriptase kit (Takara). The expression levels of several important genes in the JA biosynthesis pathway were detected, including the following: *LOX6* (forward primer sequence (5′–3′): GCTTGAAATCAATGCTCGTGCG; reverse primer sequence (5′–3′): GTCAATCACAAGCCTCACGCCAC), *MYC2* (forward primer sequence (5′–3′): GATGAGGAGGTGACGGATACGGAA; reverse primer sequence (5′–3′): CGCTTTACCAGCTAATCCCGCA), *JAZ4* (forward primer sequence (5′–3′): TCTCCGATAACTGTGAAGGA; reverse primer sequence (5′–3′): CTTGCTGACTGGAACCTG), *JAZ8* (forward primer sequence (5′–3′): AATTGTGACTTGGAACTTCG; reverse primer sequence (5′–3′): TTGCCTGAAGATGGGTAAC); *PDF1.2* (forward primer sequence (5′–3′): CCATCATCACCCTTATCTTCGC; reverse primer sequence (5′–3′): TGTCCCACTTGGCTTCTCG), *THI2.1* (forward primer sequence (5′–3′): GTTCGTATACGTGAAGGGAGTA; reverse primer sequence (5′–3′): CACACACTACATATTATCGACT), *VSP1* (forward primer sequence (5′–3′): CGTATCCGTCACCTCCTCAT; reverse primer sequence (5′–3′): CGCCAAAGGACTTGCCCTAA), *VSP2* (forward primer sequence (5′–3′): GGCCTTGCATCTTTACCAAAAC; reverse primer sequence (5′–3′): GTAGTAGAGTGGATTTGGGAGC). *ACTIN2* (forward primer sequence (5′–3′): AGTGGTCGTACAACCGGTATTGT; reverse primer sequence (5′–3′): GATGGCATGAGGAAGAGAGAAAC) and *EF1α* (forward primer sequence (5′–3′): TCCAGCTAAGGGTGCC; reverse primer sequence (5′–3′): GGTGGGTACTCGGAGA) were used as references [34,35,36,37,38]. qPCR was performed in a 7500 fast real-time PCR system (Applied Biosystems, Foster City, CA, USA) using a Power SYBR Green PCR Master Mix kit (Applied Biosystems, Foster City, CA, USA), and the 2^−ΔΔCt^ method was used to calculate relative gene expression levels [39].

### 4.13. Statistical Analysis

Student’s *t*-test and Dunnett’s C (variance not neat) at the level of *p* < 0.05 was significant. Error bars denote ± standard error of mean (SEM).

### 4.14. Accession Numbers

The GenBank numbers and Genome Initiative numbers of all genes used in this article are as follows: RBOHD (GenBank NM_124165, AT5G47910), TPC1 (GenBank NM_116594, AT4G03560), CAM3 (GenBank NM_115539, AT3G56800), ACA8 (GenBank NM_001345242, AT5G57110), LOX6 (GenBank NM_105423, AT1G67560), MYC2 (GenBank NM_102998, At1G32640), JAZ4 (GenBank NM_001123978, AT1G48500), JAZ8 (GenBank NM_102753, AT1G30135), PDF1.2 (GenBank NM_123809, AT5G44420), THI2.1 (GenBank NM_105885, AT1G72260), VSP1 (GenBank NM_001125801, AT5G24780), VSP2 (GenBank NM_001036860, AT5G24770), ACTIN2 (GenBank NM_001338358, AT3G18780), EF1α (GenBank NM_001125992.1, AT5G60390).

## 5. Conclusions

In this study, we revealed the early signaling process induced by linalool. H_2_O_2_ burst mainly because of NADPH oxidase RBOHD, then calcium ions were released into the cytoplasm through intracellular calcium stores, and the excess calcium ions in the cytoplasm were pumped out through the interaction between CAM3 and ACA8. In addition, linalool may enhance insect resistance by activating the JA pathway in Arabidopsis plants, which requires further study.

## Data Availability

The data that support the findings of this study are available from the corresponding author upon request.

## Supplementary Materials

The following supporting information can be downloaded at: https://www.mdpi.com/article/10.3390/ijms23105357/s1.

## References

[1] Kiep V., Vadassery J., Lattke J., Maaß J., Boland W., Peiter E., Mithöfer A. (2015). Systemic cytosolic Ca^2+^ elevation is activated upon wounding and herbivory in Arabidopsis. New Phytol..

[2] Ranf S., Eschen-Lippold L., Pecher P., Lee J., Scheel D. (2011). Interplay between calcium signalling and early signalling elements during defence responses to microbe- or damage-associated molecular patterns. Plant J..

[3] Kudla J., Batistic O., Hashimoto K. (2010). Calcium Signals: The Lead Currency of Plant Information Processing. Plant Cell.

[4] Ozawa R., Bertea C.M., Foti M., Narayana R., Arimura G., Muroi A., Horiuchi J., Nishioka T., Maffei M.E., Takabayashi J. (2009). Exogenous polyamines elicit herbivore-induced volatiles in lima bean leaves: Involvement of calcium, H_2_O_2_ and Jasmonic acid. Plant Cell Physiol..

[5] Zebelo S.A., Maffei M.E. (2015). Role of early signaling events in plant-insect interactions. J. Exp. Bot..

[6] Gilroy S., Suzuki N., Miller G., Choi W., Toyota M., Devireddy A.R., Mittler R. (2014). A tidal wave of signals: Calcium and ROS at the forefront of rapid systemic signaling. Trends Plant Sci..

[7] McCormack E., Tsai Y., Braam J. (2005). Handling calcium signaling: Arabidopsis CaMs and CMLs. Trends Plant Sci..

[8] Zhou S., Jia L., Chu H., Wu D., Peng X., Liu X., Zhang J., Zhao J., Chen K., Zhao L. (2016). Arabidopsis CaM1 and CaM4 Promote Nitric Oxide Production and Salt Resistance by Inhibiting S-Nitroso glutathione Reductase via Direct Binding. PLoS Genet..

[9] Chu M., Li J., Zhang J., Shen S., Li C., Gao Y., Zhang S. (2018). AtCaM4 interacts with a Sec14-like protein, PATL1, to regulate freezing tolerance in Arabidopsis in a CBF-independent manner. J. Exp. Bot..

[10] Zhang W., Zhou R., Gao Y., Zheng S., Xu P., Zhang S., Sun D. (2009). Molecular and genetic evidence for the key role of AtCaM3 in heat-shock signal transduction in Arabidopsis. Plant Physiol..

[11] Liu H., Sun D., Zhou R. (2005). Ca^2+^ and AtCaM3 are involved in the expression of heat shock protein gene in Arabidopsis. Plant Cell Environ..

[12] Tidow H., Poulsen L.R., Andreeva A., Knudsen M., Hein K.L., Wiuf C., Palmgren M.G., Nissen P. (2012). A bimodular mechanism of calcium control in eukaryotes. Nature.

[13] Dudareva N., Klempien A., Muhlemann J.K., Kaplan I. (2013). Biosynthesis, function and metabolic engineering of plant volatile organic compounds. New Phytol..

[14] Heil M. (2014). Herbivore-induced plant volatiles: Targets, perception and unanswered questions. New Phytol..

[15] Piesik D., Wenda-Piesik A., Krasińska A., Wrzesińska D., Delaney K.J. (2016). Volatile organic compounds released by Rumex confertus following Hypera rumicis herbivory and weevil responses to volatiles. J. Appl. Entomol..

[16] Yang T., Stoopen G., Thoen M., Wiegers G., Jongsma M.A. (2013). Chrysanthemum expressing a linalool synthase gene ‘smells good’, but ‘tastes bad’ to western flower thrips. Plant Biotechnol. J..

[17] Taniguchi S., Hosokawa-Shinonaga Y., Tamaoki D., Yamada S., Akimitsu K., Gomi K. (2014). Jasmonate induction of the monoterpene linalool confers resistance to rice bacterial blight and its biosynthesis is regulated by JAZ protein in rice. Plant Cell Environ..

[18] Raguso R.A. (2016). More lessons from linalool: Insights gained from a ubiquitous floral volatile. Curr. Opin. Plant Biol..

[19] Ginglinger J., Boachon B., Höfer R., Paetz C., Köllner T.G., Miesch L., Lugan R., Baltenweck R., Mutterer J., Ullmann P. (2013). Gene coexpression analysis reveals complex metabolism of the monoterpene alcohol linalool in Arabidopsis flowers. Plant Cell.

[20] Pragadheesh V.S., Chanotiya C.S., Rastogi S., Shasany A.K. (2017). Scent from Jasminum grandiflorum flowers: Investigation of the change in linalool enantiomers at various developmental stages using chemical and molecular methods. Phytochemistry.

[21] Mei X., Liu X., Zhou Y., Wang X., Zeng L., Fu X., Li J., Tang J., Dong F., Yang Z. (2017). Formation and emission of linalool in tea (*Camellia sinensis*) leaves infested by tea green leafhopper (*Empoasca* (*Matsumurasca*) *onukii* Matsuda). Food Chem..

[22] Ye L., Yang P., Zeng Y., Li C., Jian N., Wang R., Huang S., Yang R., Wei L., Zhao H. (2021). Rhizobium Symbiosis Modulates the Accumulation of Arsenic in Medicago truncatula via Nitrogen and NRT3.1-like Genes Regulated by ABA and Linalool. J. Hazard. Mater..

[23] Sharma D.K., Andersen S.B., Ottosen C., Rosenqvist E. (2015). Wheat cultivars selected for high Fv/Fm under heat stress maintain high photosynthesis, total chlorophyll, stomatal conductance, transpiration and dry matter. Physiol. Plant..

[24] Maffei M.E., Mithöfer A., Boland W. (2007). Before gene expression: Early events in plant–insect interaction. Trends Plant Sci..

[25] Yan C., Fan M., Yang M., Zhao J., Zhang W., Su Y., Xiao L., Deng H., Xie D. (2018). Injury Activates Ca^2+^/Calmodulin-Dependent Phosphorylation of JAV1-JAZ8-WRKY51 Complex for Jasmonate Biosynthesis. Mol. Cell.

[26] Chen Y., Cao C., Guo Z., Zhang Q., Li S., Zhang X., Gong J., Shen Y. (2020). Herbivore exposure alters ion fluxes and improves salt tolerance in a desert shrub. Plant Cell Environ..

[27] Asai N., Nishioka T., Takabayashi J., Furuichi T. (2009). Plant volatiles regulate the activities of Ca^2+^-permeable channels and promote cytoplasmic calcium transients in Arabidopsis leaf cells. Plant Signal. Behav..

[28] Guo J., Zeng W., Chen Q., Lee C., Chen L., Yang Y., Cang C., Ren D., Jiang Y. (2016). Structure of the voltage-gated two-pore channel TPC1 from Arabidopsis thaliana. Nature.

[29] Pottosin I., Wherrett T., Shabala S. (2009). SV channels dominate the vacuolar Ca^2+^ release during intracellular signaling. FEBS Lett..

[30] Huda K.M.K., Banu M.S.A., Tuteja B., Tuteja N. (2013). Global calcium transducer P-type Ca-ATPases open new avenues for agriculture by regulating stress signalling. J. Exp. Bot..

[31] Loreto F., D’Auria S. (2022). How do plants sense volatiles sent by other plants?. Trends Plant Sci..

[32] Scholz S.S., Vadassery J., Heyer M., Reichelt M., Bender K.W., Snedden W.A., Boland W., Mithöfer A. (2014). Mutation of the Arabidopsis Calmodulin-Like Protein CML37 Deregulates the Jasmonate Pathway and Enhances Susceptibility to Herbivory. Mol. Plant.

[33] Vadassery J., Reichelt M., Hause B., Gershenzon J., Boland W., Mithöfer A. (2012). CML42-Mediated Calcium Signaling Coordinates Responses to Spodoptera Herbivory and Abiotic Stresses in Arabidopsis. Plant Physiol..

[34] Mustilli A., Merlot S., Vavasseur A., Fenzi F., Giraudat J. (2002). Arabidopsis ost1 protein kinase mediates the regulation of stomatal aperture by abscisic acid and acts upstream of reactive oxygen species production. Plant Cell.

[35] Brown R.L., Kazan K., McGrath K.C., Maclean D.J., Manners J.M. (2003). A role for the GCC-Box in jasmonate-mediated activation of the *PDF1.2* gene of Arabidopsis. Plant Physiol..

[36] Gutierrez L., Mauriat M., Guénin S., Pelloux J., Lefebvre J., Louvet R., Rusterucci C., Moritz T., Guerineau F., Bellini C. (2008). The lack of a systematic validation of reference genes: A serious pitfall undervalued in reverse transcription-polymerase chain reaction (RT-PCR) analysis in plants. Plant Biotechnol. J..

[37] Yan S., Luo S., Dong S., Zhang T., Sun J., Wang N., Yao H., Shen Y. (2015). Heterotrimeric G-proteins involved in the MeJA regulated ion flux and stomatal closure in Arabidopsis thaliana. Funct. Plant Biol..

[38] Yan S., Jiao C., McLamore E.S., Wang N., Yao H., Shen Y. (2017). Insect Herbivory of Leaves Affects the Auxin Flux along Root Apices in Arabidopsis thaliana. J. Plant Growth Regul..

[39] Livak K.J., Schmittgen T. (2001). Analysis of relative gene expression data using real-time quantitative PCR and the 2^−ΔΔCt^ method. Methods.

