# Supplementary material for: Linalool Activates Oxidative and Calcium Burst and CAM3-ACA8 Participates in Calcium Recovery in Arabidopsis Leaves
Source: Int J Mol Sci. 2022 May 11;23(10):5357. doi: 10.3390/ijms23105357 (PMC9142083; doi:10.3390/ijms23105357)
Supplement: Supplementary file 1 [file ijms-23-05357-s001.zip › ijms-1717187-supplementary.pdf]

Supplementary Materials

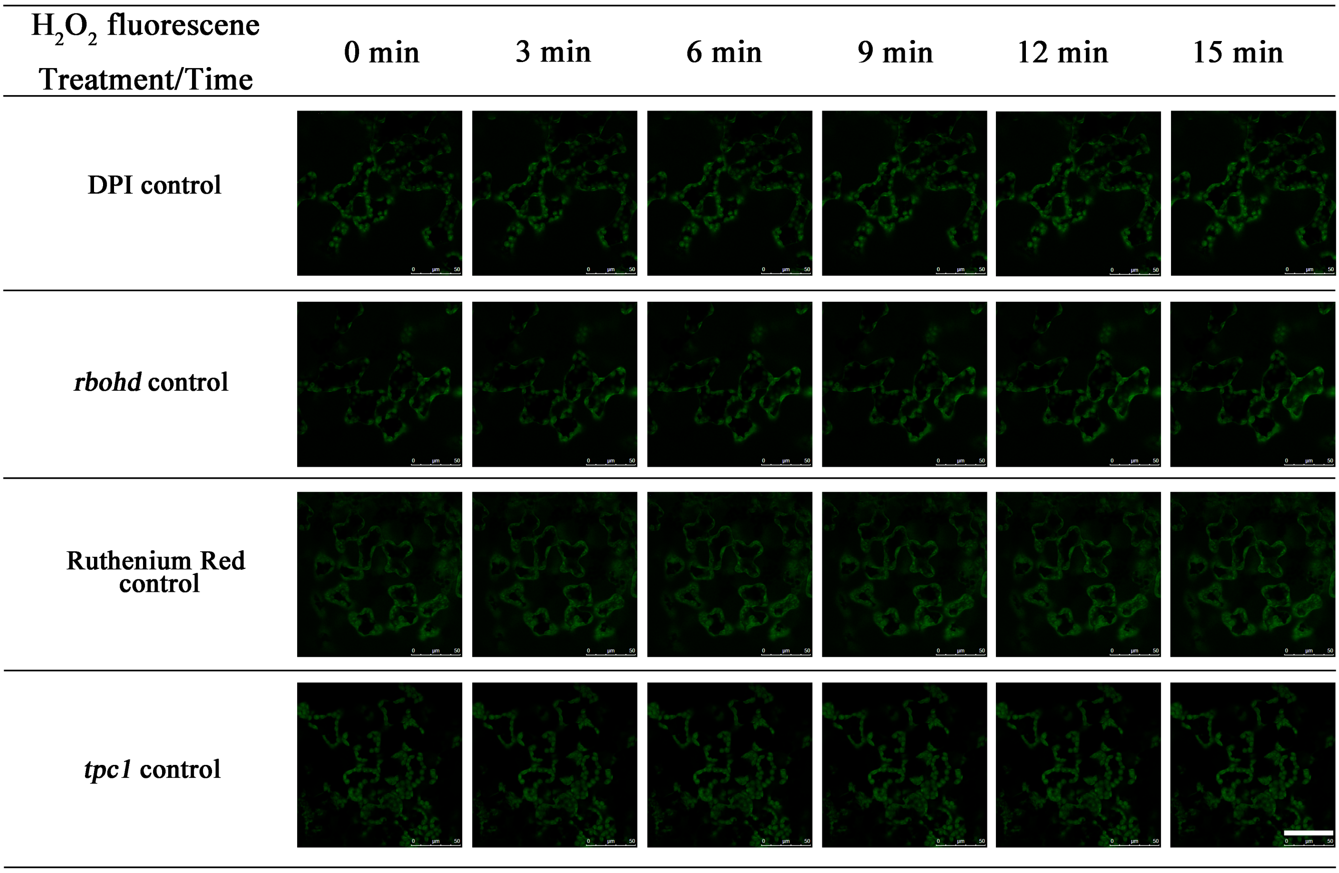

Figure S1. H_2O_2 fluorescence in the control groups. The scale bar represents 50 μm.

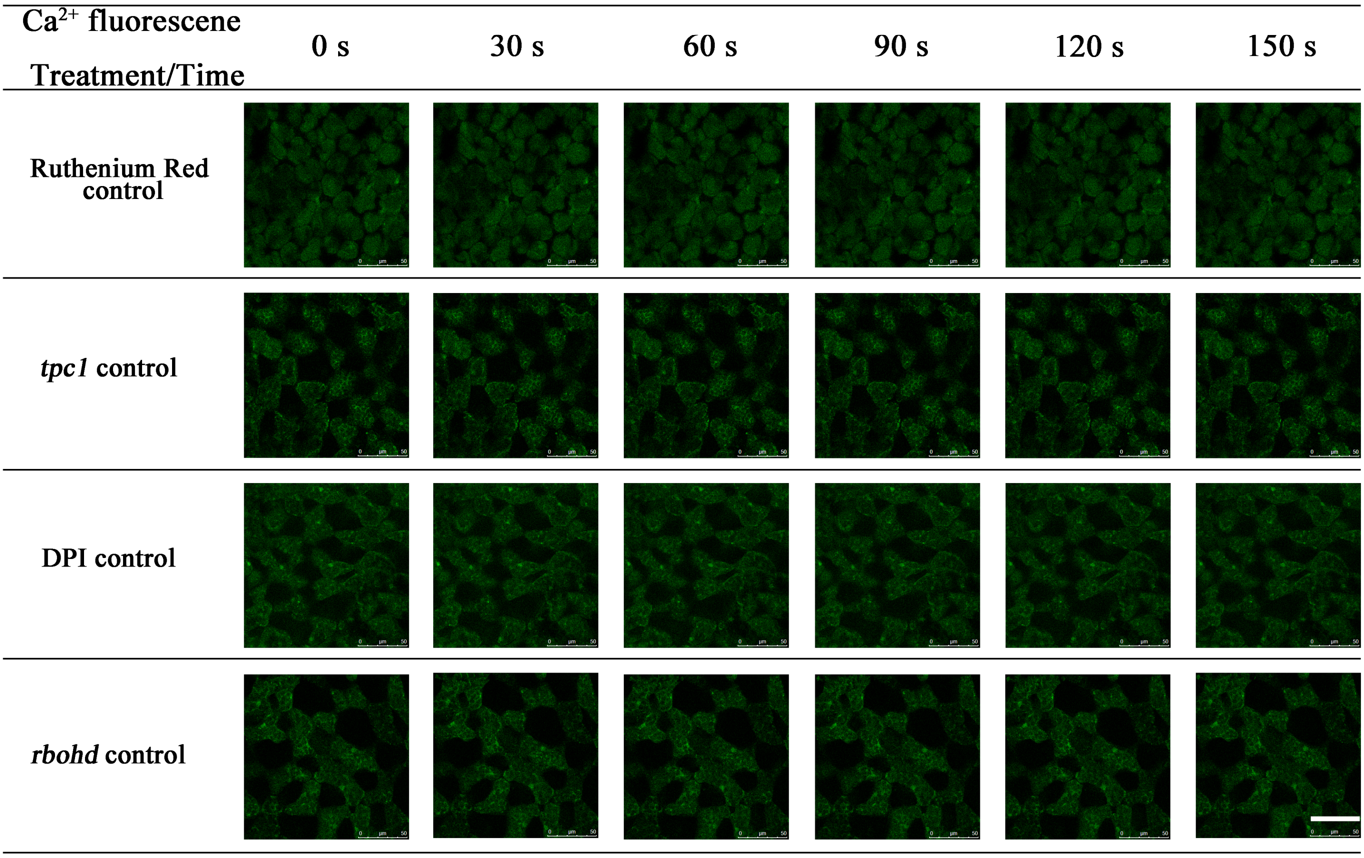

Figure S2. Ca^{2+} fluorescence in the control groups. The scale bar represents 50 μm.
